# Chairman’s Address

**Published:** 1896-03

**Authors:** M. H. Fletcher

**Affiliations:** Cincinnati, O.


### THE DENTAL REGISTER.

VOL. L.] MARCH, 1896. [No. 3.

### COMMUNICATIONS.

### Chairman's Address.

BY M. H. FLETCHER, M.D., D.D.S., CINCINNATI, O.

Delivered in the Section on Dental and Oral Surgery, at the Forty-sixth Annual Meeting of the American Medical Association, at Baltimore, Md., May 7-10, 1895.

I acknowledge with pleasure the courtesy shown me, in again selecting me as presiding officer; though this Section is not large as a national body, the quality of its work is second to none, and its influences are unbounded. Its tendency is to elevate to a higher standard thereby advancing the science of the profession. In looking back over the past twelve months, these marks of progress are encouragingly conspicuous and, we believe, point to a steady and healthy growth. This is indicated by the increased number of practical scientific workers who are coming forward; by books of scientific value second to none in their line; by numbers of good papers and discussions on important topics, many of which have served largely to add to our fund of knowledge. Errors have been eliminated which is equally important with new discoveries. Superstition and guessing are giving place to accuracy and precision.

It is a fact that all the professions which have man himself for the subject, which aim to rectify his physical deficiencies, are to-day, and always have been, of the greatest interest; their scientific advancements in the past decade are more marked than in any previous one, but our *lack* of knowledge is still very great.

Through the kindness of nature in maintaining her laws, regardless of all theories or methods, our patients recover and are even grateful, believing the result to be in exact accord with the laws which we are supposed to understand; but though we may have been both honest and conscientious in our work, does this lessen our responsibility? In other words, should we not, and can we not, know better than we do? And do we take all means accessible to inform ourselves?

When we work or reason from wrong principles, nature keeps coming back at us with the same conditions, and by continuous knocking at our obtuseness, tries to show us the right way; and when at last we perceive and accept her teachings, we become, through our enlightenment, the true benefactors of mankind, and I take it that there is no nobler nor happier position in which man may find himself. Now, in order that we may attain this desirable position, our minds must be constantly on the alert, willing to correct errors, and sensitive to new truths, much as the photographer's plate is sensitive to light and shadow. This might seem a dry and monotonous pastime, but those who have tried it longest have gained the most pleasure from life, and have been of greatest usefulness. Lubbock says: "Those who have not tried for themselves, can hardly imagine how much science adds to the interest and variety of life." This kind of life and work gives us all the poetry we many desire with a limitless field for imagination; it gives us art, for nature is art; it gives us history, for facts about people and nations is history; it gives us science, for knowledge of the laws of the universe is science. Nor is this the limit, for after all our seeking there yet remain truths that are beyond the mind of man to conceive.

I am aware that when I touch upon the best methods of educating students for our profession, I run the risk of being prosy and tiresome; but the subject bears so directly on our standing in the future, that I presume upon your courtesy to the chair, and take advantage of my opportunity. Discussion of this point has not been confined to our specialty alone. Educators in almost every line have been much agitated over the same problem. I am persuaded from what I have read and observed on this topic, that a good way to try to solve the question is through the study of mental philosophy, the laws of which within certain limits are as unchangeable as those pertaining to the physical man. Now if we take into consideration the fact that it is through sense-perception that we receive all our knowledge, would it not seem that the technique classes or laboratory methods would be the most natural channel through which to educate not only our students, but those in all branches of medicine? Recall for instance the delicate technique required of the successful surgeon, the oculist, the throat specialist or gynecologist; even the general practitioner needs a gentle and educated touch, and a most thoroughly-trained mind in reasoning from cause to effect, and effect to cause. Good and accurate results come only to him who has learned how to reason after this manner, first on the simplest matters in philosophy; then gradually going on, step by step, until he is able to handle in a practical way the more obtruse problems. This training begins in babyhood, from the time the infant first notices the candle-light, and by experiment learns that if he touches it, he suffers pain. Thus he accumulates one fact after another, until he finally reaches the last stages of mental development, that of judgment and ideation. In connection with this subject of mental ripening comes the question, which has been somewhat discussed, as to whether a student showing no adaptability in learning the branches he has chosen, should be allowed to continue at such work; or whether he should be shown his defects, and encouraged to try some branch of work for which he may have some capabilities. Pertinent to this point, there is in the February number of *The Educational Review*, an article on "The Education of the Nervous System," by Henry H. Donaldson, in which he says: "No amount of education will cause enlargement or organization where the rough materials, the cells, are wanting; and on the other hand where these materials are present, they will in some degree become evident, whether purposely educated or not;" he further says: "On neurologic grounds, therefore, nurture is to be considered of much less importance than nature, and in that sense the capacities that we most admire in persons worthy of remark are certainly born rather than made."

Again he says, in speaking of early mental ripening or precocity: "The same conditions which give the individual a generously-planned nervous system also favors its early development." In such precocious persons it tends to grow for a longer period than usual, a feature which is fully as important as the precocity itself. It is extremely interesting to see how, among a series of eminent men, excluding men of action, the determination of distinction follows the order in which the brain normally attains the high development necessary to command recognition in a particular profession. He gives the following table based on 287 cases analyzed by Prof. James Sully:

| 287 Cases analyzed by Prof. Sully. | Gave Promise Before 20 Years Old. | Produced Before 30 Years Old. | Attained Distinction Before 40 Years Old. |
|---|---|---|---|
| Music'ans........ | 95 per cent. | 100 per cent. | 100 per cent. |
| Artists............ | 89 per cent. | 98 per cent. | 100 per cent. |
| Scholars.......... | 83 per cent. | 71 per cent. | 90 per cent. |
| Poets.............. | 75 per cent. | 92 per cent. | 92 per cent. |
| Scientists ......... | 75 per cent. | 80 per cent. | 92 per cent. |
| Novelists......... | 75 per cent. | 56 per cent. | 80 per cent. |
| Philosophers...... | 67 per cent. | 56 per cent. | 60 per cent. |

"Those professions demanding only small acquisition but a very perfect adjustment between one sense organ, and one set of muscles, as between the hand and the ear in the musician, and the eye and the hand in the artist, are precocious throughout, while the philosophers with their need for accumulated information and ripened judgment bring up the rear. Similar investigations on slightly different material yield accordant results."

The art of our profession would naturally compare with the artist in the table, for success in this department is dependent upon the training and skill of the eye and the hand; again, the science of the profession compares with the scientists in the table, who come much lower in the list, showing the necessity of more strength of mind, and a greater accumulation of facts. But to be simply an artisan and a scientist is not sufficient to enable one to reach the highest standard of usefulness as a practitioner of medicine or its specialties. In our own division one needs to be somewhat proficient in at least four callings, namely: As an artist, a scholar, a scientist and a philosopher. If one of fair ability honestly endeavors to meet the demands of his profession in these four callings, all things desirable will be at his command.

It must be evident that it matters not whether a student be dull or precocious, the amount of his knowledge is proportional to his accumulation of facts and his ability to recall and utilize them. Different men have different abilities. Some persons do with great ease or almost intuitively those things that are only done with the greatest effort by others, if they succeed at all. This being the case, the standard of license to practice any department of medicine should be so guarded that no incompetent person could enter its ranks. As to the best methods of educating for this standard, the most natural, as before stated, is that method in which the senses convey knowledge by contact with the objects and phenomena in actual work, at first in its simplest form, then gradually leading step by step to perfection; by this method the mind is stored with facts and trained to reason, and use them to the end desired.

The "technic system" of teaching was very ably presented last May by Dr. Edward C. Kirk, before the Academy of Dental Science of Boston and well discussed by its members, and I wish to say that I heartily agree with the essayist. There is, however, one feature of education in both dental and medical colleges, which was only touched upon in the paper and mentioned by no one during its discussion, yet I consider it a question of the greatest importance. It is that of having suitably-trained instructors in our colleges. This I deem to be as great a defect as any at the present time in our system of teaching.

At the last meeting of the American Dental Association during the discussion of the report of the Committee on Dental Education, Dr. H. J. McKellops struck the keynote when he said: "Give me the man who can teach science." If a student is bright and apt in his college work he will make good progress under almost any circumstances, but if he makes good progress with poor instruction, how much better would he do under thoroughly-trained teachers. It has been my pleasure to be a student, under instruction, in some branch almost continuously for the past eighteen years, and this feature of ability, or lack of it, to impart knowledge is a point that has impressed me very strongly with every change of subject and instructor. It is not every one that has knowledge who knows how to impart it intelligently, and the fewer number knows how to present a subject in a form most suited to the mental status of their hearers. If the capabilities of all students were equal, and their interest in progress were the same, the matter would not present so many difficulties to the instructor, but were these conditions possible, the same class would progress much more rapidly under one teacher than under another. An article in a recent magazine by a college student compares two professors in the following manner: "Though he thoroughly knows his subject, his instruction is about as clear as mud; with him everything is 'perfectly obvious,' but to us his attempts at exposition are positively confusing; he thinks only of his subject, never of his students; he is cold, unsympathetic, unapproachable. I want to know the nature of this requirement for to-morrow, but if I ask him he will either show impatience at my ignorance or temerity, or misconstrue the motive of my inquiry and stand upon his pedestal of superior knowledge. But with Professor X. we make headway; he seems anxious and able to make everything clear; he puts himself in his pupil's place; he is always willing to remain after the lecture to answer questions or discuss debatable points."

Charles C. Ramsay, in the January number of the *Educational Review*, speaks of this subject as follows: "True teaching, whether in school or college, is the process of causing another to know through self activity, and includes the mutual effort of two persons to the same end, the teacher and the learner. Until the two are at this common work the process of teaching has not been begun; until the learner has learned, the teacher has not taught." And let it be remembered that the proof of the teaching process always rests with the learner, not with the teacher, whether the scholars be young or old. "The teacher can prove that he tried to teach; the scholar alone can show that the teacher succeeded." "The measure of information," said Pestalozzi, "is not what the teacher can give, but what the pupil can receive."

These fundamental principles of all good teaching are as applicable to collegiate as to elementary and secondary instruction.

Now, if this be the case in literary institutions and universities, where the professors and teachers give their entire time to this work, and should know how to do it in the best possible manner, how much more is it true in dental and medical colleges, where the teaching is done by men whose time is almost wholly taken up by a busy practice; many of them are eminent and skilled in their professional duties, yet are quite marked for their lack of ability to teach; nevertheless, their students are expected to reach a high standard under their tutorage. Is it any wonder then that students pony, and resort to all kinds of methods for getting assistance at examinations, under such conditions?

The subject as to whether the lecture method or that of recitation is best has been pretty fully discussed amongst our educators, but whichever method is adopted it in no way makes a good or bad teacher. Now, if one needs so much training in order to properly fill a simple cavity or extract a tooth, a procedure which seems so simple to us, how much greater his obligation to be properly prepared before attempting to direct and instruct the intellect of others. The loss of teeth or a limb may be replaced by artificial substitutes, but the loss of time can never be replaced; and time to many students is as valuable as to the professor; probably not from an immediate financial standpoint, but the loss of time to an earnest, intelligent student at an age when he is studying a profession is not a trivial matter. You may say that many students, or even most of them, care but little for their time, or what they learn, so they are graduated and given a license to practice; granting this to be true, it is all the greater reflection upon their instructors, for if the work was made more interesting the number of attentive students would increase proportionally and develop into the kind of practitioner we delight to recognize; it is a known fact that it is not always the pupils who stand highest in a class that shows the best results in after life; the plodding, honest ones in almost every class many times outnumber the precocious ones; and under the guidance of properly-trained and intelligent instructors, the number of honest plodders might be increased from the ranks of those who are apparently dull and careless, and the list of precocious might be enlarged from those who are brilliant and capable, but who fritter away their time in gaiety and foolishness. It is not an uncommon thing to find in a class students who outrank many of their professors in strength of intellect and natural ability; not only these, but all students have a right to demand that their instruction be presented to them in the best possible manner.

It is a perfectly easy matter to get professors for the asking, but trained instructors are rare; and even those who have the natural qualities to become good instructors are not numerous. No one doubts that we have professors in almost every college who are thoroughly capable, and who take time and pains to be what they should to the student, but, on the other hand, a greater number fall far short of this standard.

I believe a majority of students in all colleges are industrious and of good intent, and many of them are barely able, financially, to carry through a three or four years' course; such students, if denied the full measure of their capacities to receive, are defrauded of both money and time, the first of which never is, and the last never can be repaid. There are no normal schools, so far as I know, for the proper training of dental and medical teachers, and if there were, it does not seem practicable for many of our eminent practitioners, who are in other respects most suited for the position of instructors, to leave work and go to other localities for such training; but such a normal school, it seems to me, is demanded in some suitable form, and it would seem a most fitting topic for discussion and action by our national associations of faculties. The distance a missile flies and the velocity with which it goes is exactly proportional to the force which impels it. The analogy holds good in our colleges, for students of high grade can not be graduated unless the force can be found in their Alma Mater to produce the desired result; consequently, it would seem no more necessary, if as much, to lengthen the term at college, increase the curriculum, and raise the standard of entrance, than it is to increase the quality and ability of college instructors.

